# Study on the Bending–Shear Properties of Concrete-Filled Circular Carbon Fibre Reinforced Plastic Steel Tubes

**DOI:** 10.3390/ma17122895

**Published:** 2024-06-13

**Authors:** Qingli Wang, Haiyu Qin, Kuan Peng

**Affiliations:** 1 School of Civil Engineering, University of Science and Technology Liaoning, Anshan 114051, China; 2 School of Intelligent Manufacturing, Chengdu Technological University, Chengdu 610031, China

**Keywords:** circular CFRP steel tube, in-filled concrete, bending–shear behavior, numerical study, bearing capacity

## Abstract

In order to study the bending–shear performance of CFRP concrete-filled steel tubes, static tests were conducted on 15 circular concrete-filled CFRP steel tube bending–shear specimens. For all specimens, *D*_s_ was 120 mm, *t*_s_ was 2 mm, and *m*_l_ was 1. The shear displacement (*V*-Δ) curve of the specimen and the collaborative work between the steel tube and CFRP are discussed. ABAQUS was applied to simulate the *V*-Δ curve and failure mode of the specimen. We explored the effects of CFRP layers, material strength, the steel ratio, and the shear span ratio on the bending–shear performance of components. The experimental results show that a steel tube and CFRP can work together. As the shear span ratio increased, the bearing capacity and stiffness of the specimen decreased. An increase in the number of transverse CFRP layers could improve the bearing capacity of the specimen, but it had no significant effect on the stiffness. Calculating the elastic stage stiffness and bearing capacity of 15 short columns of test and FE curves revealed an average error of 6.71% and a mean square error of 0.83 for the elastic stage stiffness. The simulation results of the established finite element model are in good agreement with the experimental results. The average error of the bearing capacity was 3.88%, with a mean square error of 0.94. Based on experimental and finite element results, the moment shear correlation equation for concrete-filled CFRP steel tube bending–shear members is presented.

## 1. Introduction

The term CFRP concrete-filled steel tube refers to a structure formed by pouring concrete into CFRP steel composite tubes [1,2,3]; it also refers to a structure formed by reinforcing or repairing concrete-filled steel tubes with CFRP [4,5,6]. The existence of CFRP can improve the durability of concrete-filled steel tubes, and CFRP can also delay the buckling deformation of steel tubes. Furthermore, in terms of its mechanical properties, CFRP has high tensile strength; so, for concrete, in addition to the constraints of steel tubes, CFRP can also provide good constraint effects and improve its mechanical properties. In engineering practice, structural members mainly bear axial loading, bending moment, compression bending, and other loads, but sometimes, the bending–shear force cannot be ignored—for example, in stub columns supporting spatial latticed shells, piers with large section sizes, nodes with slant supports, etc., and structures under transverse loads such as those caused by earthquakes, blasts, and impacts can also be subject to higher bending–shear loads [7,8,9,10,11].

Many research results have been presented on CFRP concrete-filled steel tubes, including experimental research, finite element simulations, theoretical analyses, bearing capacity calculation/related equations, and restoring force models. Yu et al. [12] analyzed existing experimental data and proposed a calculation expression for the bearing capacity of FRP concrete-filled steel tubes. Research has found that the mechanical characteristics of FRP concrete-filled steel tubes mainly depend on the equivalent constraint coefficient before FRP failure [13]. Tao et al. [14] conducted experiments on the bending performance of CFRP-reinforced, concrete-filled steel tubes after a fire; the experimental results indicated that the repair effect of CFRP on flexural specimens is not as good as that on axially compressed short columns, perhaps due to the absence of longitudinal CFRP. Yu et al. [15] established 12 numerical models using ABAQUS 6.1.14 to study the effect of the slenderness ratio on the mechanical performance of CFRP-confined, concrete-filled steel tube long columns; the analysis results indicated that as the slenderness ratio increased, the bearing capacity, stiffness, and maximum equivalent stress value of the steel tube specimen decreased, and the steel tube hoop effect weakened. Du et al. [16] conducted axial compression performance tests on rectangular high-strength, concrete-filled steel tube long columns constrained by CFRP, with experimental parameters including the slenderness ratio, the width thickness ratio of specimen cross-section, concrete strength, and the number of CFRP layers. Sundarraja et al. [17] studied the strengthening effect of CFRP on concrete-filled steel tube bending members, with a total of 18 specimens. At the same time, a nonlinear finite element model was established to verify the stress–strain relationship curve and corresponding failure modes. The research results show that some specimens pasted with CFRP were damaged due to the peeling of CFRP, and some did not even reach the corresponding bearing capacity of the steel tube concrete specimens. However, specimens pasted with CFRP along their entire lengths had significantly improved bending bearing capacities and stiffnesses. Al Mekhlafi et al. [18] conducted eccentric compression tests on 12 CFRP-restrained stainless steel-reinforced concrete short columns and established a three-dimensional finite element model for numerical simulations based on the tests. Chen et al. [19] studied the impact performance of FRP-reinforced steel tube concrete and analyzed the dynamic performance of various types of FRP-reinforced steel tube concrete with different steel tube thicknesses under lateral impact under different load conditions using a finite element model. The results indicated that FRP steel tube concrete has a higher bearing capacity and better toughness, making it more suitable for structures subjected to impact loads. Cai et al. [20] tested the stress performance of six full-scale cantilever specimens under the combined action of a constant axial force and lateral hysteresis displacement, including CFRP-reinforced concrete pipes. One GFRP tube-reinforced concrete specimen and one thin-walled steel tube-reinforced concrete specimen, as well as two GFRP thin-walled steel tube-reinforced concrete specimens were used. The experimental results indicated that GFRP limits the occurrence of the local buckling of steel pipes and the sudden failure of welds due to excessive biaxial stress. When the axial compression ratio increases from 0.2 to 0.45, the displacement ductility of GFRP steel tube concrete remains almost unchanged.

This type of structure is widely used in bridges and other structures, but there is currently limited research on the bending–shear coupling load of concrete-filled CFRP steel tubes, and there is no reasonable finite element model. In view of this, a testing device and method for structural members under bending–shear loads have been developed. We conducted relevant experimental research with the shear span ratio and CFRP layers as the main parameters to investigate the influence of these parameters on the shear–bearing capacity and stiffness of the specimen, and we proposed the use of the moment shear correlation equation for concrete-filled CFRP steel tube bending–shear members.

## 2. Experimental Research

### 2.1. Design and Material Properties

Fifteen circular concrete-filled CFRP steel tube specimens were designed to determine their bending–shearing static properties. The main parameters included the number of transverse CFRP layers, *m*_t_, and the slenderness ratio, *λ*. For all specimens, *D*_s_ was 120 mm, *t*_s_ was 2 mm, and *m*_l_ was 1. Other parameters are shown in Table 1.

The performance of the steel tube specimens is shown in Table 2. The parameters of the steel were measured by tensile tests, and the tensile tests of the steel was carried out according to Metallic materials—Tensile testing—Part 1: Method of test at room temperature (GB/T 228.1-2010) [21] The yield strength was defined as *f*_y_, the tensile strength was defined as *f*_u_, and the tensile rate was defined as *e*’. The elastic modulus was defined as *E*_s_ and the Poisson’s ratio was defined as *v*_s_.

The particle size of the coarse aggregate gravel was 5–15 mm, and a water reducer with 1% cement weight was added. The specific ratio of the concrete is shown in Table 3.

Finally, the cube compressive strength *f*_cu_ was measured from a 150 mm cube cured under the same conditions as the specimen [22]; the *f*_cu_ of the concrete for 28 d was 46.1 MPa, and the elastic modulus of the CFRP *E*_c_ was 33 GPa.

The mechanical properties of woven CFRP were measured by tensile tests; the tensile tests for woven CFRP were carried out according to test methods for the tensile properties of carbon fiber multifilaments (GB/T 3362-2017) [23]. Unidirectional woven CFRP was used as a paste on the steel tube. The main performance indexes of the woven carbon fiber are shown in Table 4. The fracture strain of transverse woven CFRP is defined as *ε*_cftr_. The fracture strain of Longitudinal woven CFRP is defined as *ε*_cflr_.

### 2.2. Loading and Measurement

Firstly, concrete filled-steel tube specimens were prepared. The steel tubes were cut according to the design dimensions, then the concrete poured, and the end plates welded after the concrete hardened. Secondly, the woven CFRP was manually pasted onto the steel, according to the specific pasting process shown as follows:

(1) An angle grinder and sandpaper were used to remove welding slag and floating rust on the surface of the steel tube. (2) Structural adhesive was applied to the surface of the concrete filled-steel tube. (3) Woven CFRP was pasted onto the surface of the steel tube. Note that the overlapping length of the two woven CFRPs was 150 mm.

**Figure 1:**
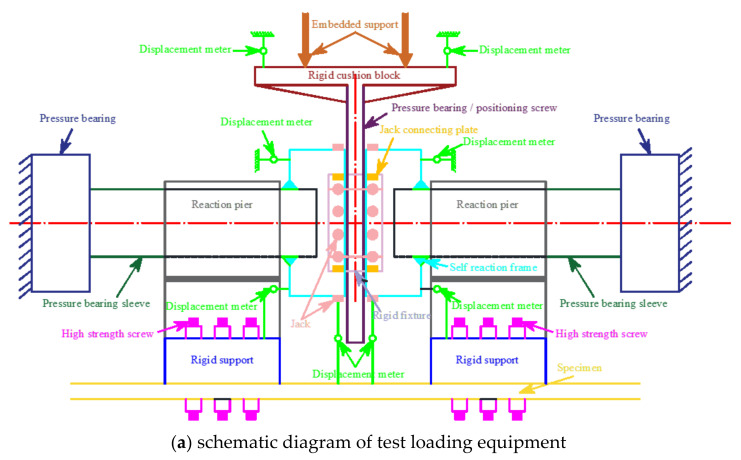

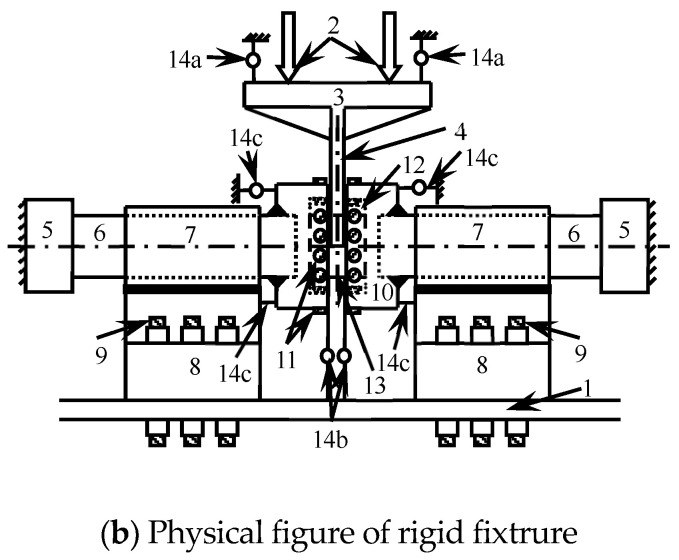
Test equipment. 1. Specimen, 2. Embedded support, 3. Rigid cushion block, 4. Pressure bearing/positioning screw, 5. Pressure bearing, 6. Pressure bearing sleeve, 7. Reaction pier, 8. Rigid support, 9. High strength screw, 10. Self reaction frame, 11. Jack, 12. Jack connecting plate, 13. Rigid fixture, 14. Displacement meter.

Specimens were prepared for testing in two steps. (1) In the elastic stage, the load of each level was about 1/10 of the estimated bearing capacity, and the load was held for 2 min [24]; (2) Loading was applied slowly and continuously until the specimen was damaged, and unloaded after the load dropped to 30% of the peak load, after entering the enhancement stage. A total of 8 displacement meters were arranged to measure the displacement (Δ) of the specimens [25]. The apparatus used in the study is depicted in Figure 1.

**Figure 2:**
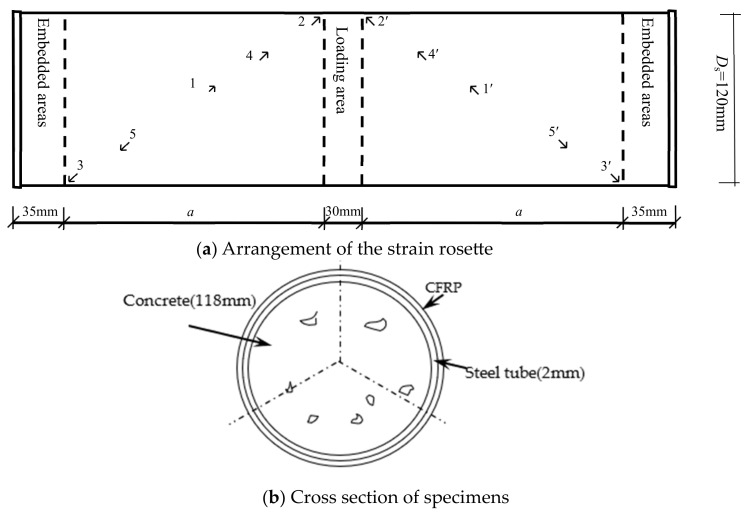
The arrangement of the strain rosette and cross section.

The arrangement of the strain rosette is shown in the Figure 2: 1 is measuring point on the steel tube; 1′ is the measuring point on the CFRP. For the specimen with *λ* = 0.15, *a* = 18 mm; For specimens with *λ* = 0.3, *λ* = 0.45, and *λ* = 0.75, *a* = 36 mm, 54 mm, and 90 mm, respectively; For the specimen with *λ* = 1.5, *a* = 180 mm.

## 3. Experimental Results

### 3.1. Failure Mode

**Figure 3:**
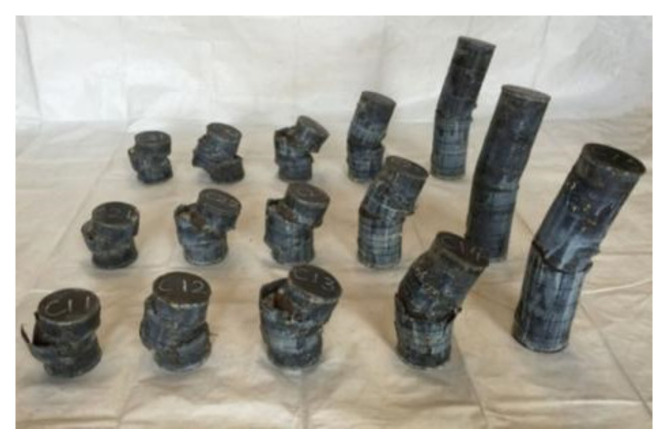
All specimens after loading.

All loaded specimens are shown in Figure 3.

According to the analysis of the test results, it was found that the test phenomena of the bending shear specimens could be divided into three types based on different shear span ratios: pure shear failure (*λ* = 0.15) [2], bending–shear failure (*λ* = 0.3~0.75), and bending failure (*λ* = 1.5) [25].

#### 3.1.1. Experimental Phenomenon of Shear Failure

**Figure 4:**
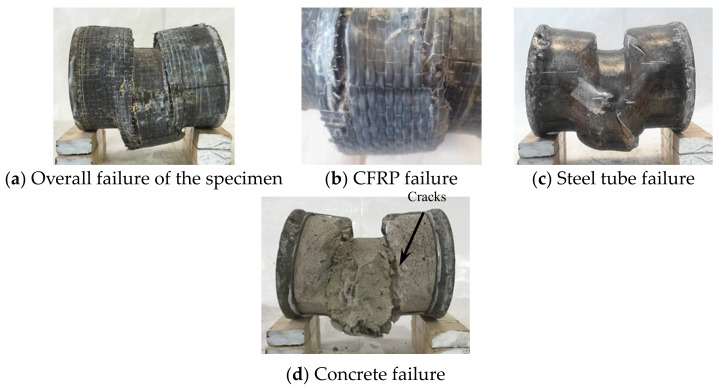
Shear failure characteristics of the specimen.

In the initial stage of loading, the relationship between the load and displacement was linear, and the specimen had no obvious deformations. When the load reached about 90% of the peak loading, the specimen in the loading area was sheared out as a whole, showing that the upper part was concave and the lower part was convex, and the CFRP fracture occurred at 1/2 of the height of the specimen section. When the peak load was reached, the steel tube at the upper part of the loading area was fractured, and the exposed concrete could be observed. As the lower steel tube was fractured along the edge of the support, the bearing capacity of the specimen began to decline and entered the softening section; with the increase of shear displacement, when the load dropped to about 65% of the peak load, a sharp popping sound could be heard, and inclined cracks appeared in the steel tube at 1/2 of the section height. After fracturing the outer tube at the end of the test, it was found that the concrete in the loading area was sheared as a whole, and inclined cracks appeared at the corresponding location to the steel tube cracks [2]. The shear failure pattern of the specimen is shown in Figure 4.

#### 3.1.2. Experimental Phenomenon of Bending Failure

**Figure 5:**
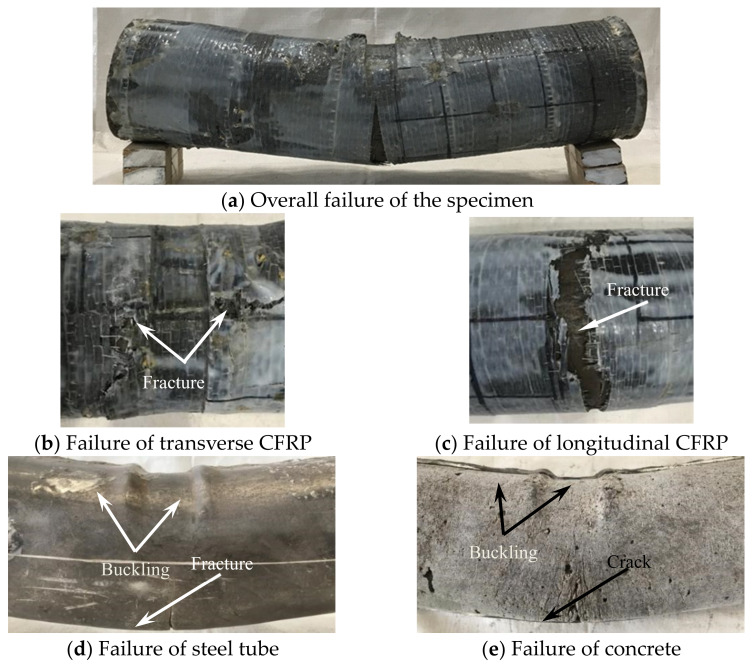
Bending failure characteristics of the specimens.

When the specimen underwent significant deformation during the later stage of loading, the transverse CFRP in the longitudinal compression zone began to fracture. The cross-section of the specimen underwent significant bending deformation. As the loading continued, the bearing capacity suddenly decreased, and the transverse CFRP began to fracture. The bending failure pattern of the circular bending specimens is shown in Figure 5.

#### 3.1.3. Experimental Phenomenon of Bending Shear Failure

**Figure 6:**
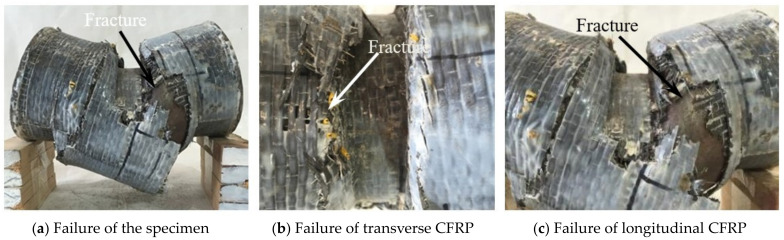
Bending shear failure characteristics of the specimens.

According to the experimental phenomenon, bending shear failure occurred in the circular bending shear specimens with λ = 0.3, λ = 0.45, and λ = 0.75. At the initial stage of loading, there was no significant deformation of the specimen, and the shear force was proportional to the displacement. When the shear force reaches about 80% of the peak shear force, the sound of continuous cracking of the adhesive can be heard. When the shear force reaches about 90% of the peak shear force, the longitudinal CFRP was pulled off in the lower tensile zone, and the shear force suddenly decreased. The transverse CFRP was cut off at the fixture or pulled off in the compression zone. When the shear force dropped to about 80% of the peak shear force, the steel entered the strengthening stage, and the shear force begins to slowly rise. The specimen underwent significant bending deformation, with the upper part becoming slightly concave at the fixture and the lower part slightly convex. When the peak shear force was reached, the upper steel tube of the specimen was cut at the fixture, and the lower steel tube was cut near the embedded end. As the displacement increases, the bearing capacity decreased, and the specimen underwent significant shear deformation. When the shear span ratio was small, the failure mode of the specimen was mainly pure shear failure, while also exhibiting the characteristics of bending failure. When the shear span was relatively large, the failure mode of the specimen was mainly bending failure, while also exhibiting the characteristics of pure shear failure. This is also consistent with the bending-shear failure mode in reference [6], but the constraint effect of the CFRP caused the degree of component failure to be relatively mild [20,24]. The typical bending–shear failure characteristics of the concrete-filled CFRP steel tubes are shown in Figure 6.

**Figure 7:**
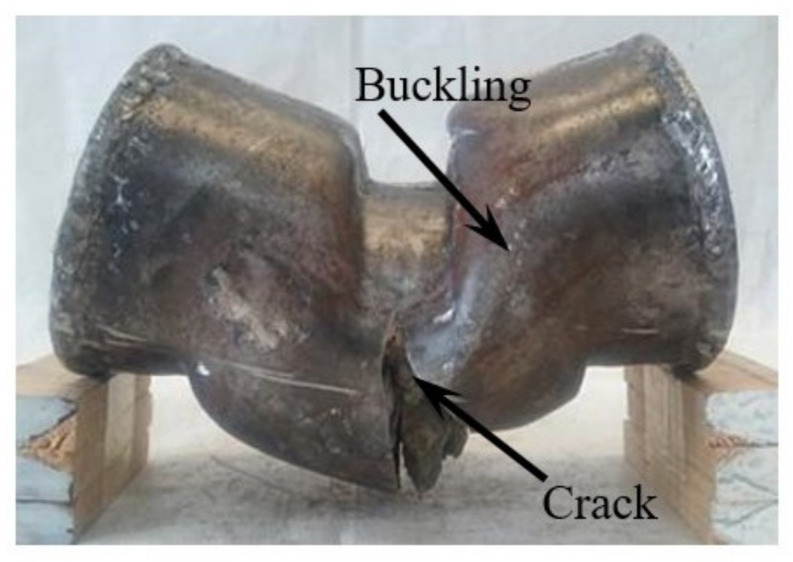
Failure characteristics of the steel tube.

Peeling off the CFRP to observe the steel tube, the failure of the upper steel tube of the specimen could be divided into two types: when the shear span ratio was small, it was cut off at the upper fixture of the specimen; when the shear span was relatively large, the steel tube only deformed and was not cut. The lower steel tube of the specimen was cut along one side of the fixture; when the shear span ratio was small, the cutting position was close to the embedded end, and when the shear span was relatively large, the fracture location was close to the loading zone (Figure 7). The steel tube near the middle section showed oblique bulging.

**Figure 8:**
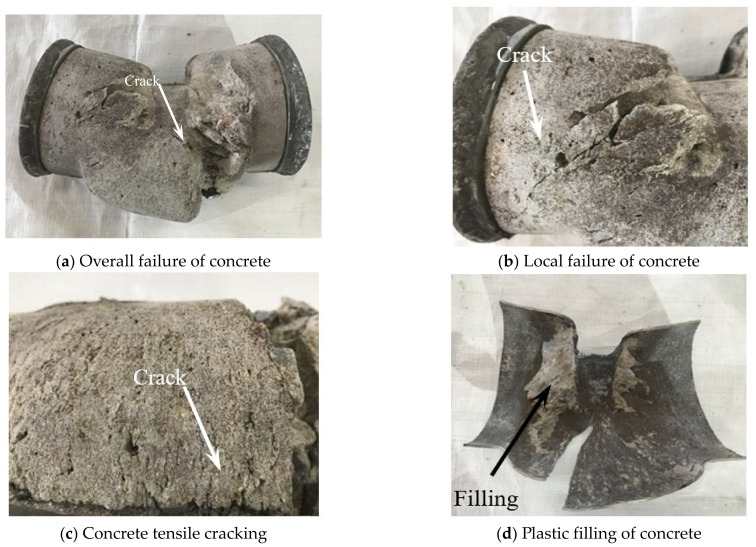
Failure characteristics of the concrete.

Cutting the steel tube, it could be seen that the concrete on the upper part of the specimen underwent shear deformation (Figure 8a). The concrete at the oblique bulging position of the steel tube produced oblique cracks (Figure 8b). The concrete in the tensile zone experienced tensile cracking (Figure 8c), and the cracks significantly increased with increases in the shear span ratio. The adhesion between the concrete and steel tubes (Figure 8d) showed good plastic filling performance.

### 3.2. Test Results and Analysis

#### 3.2.1. Shear–Displacement Curve

**Figure 9:**
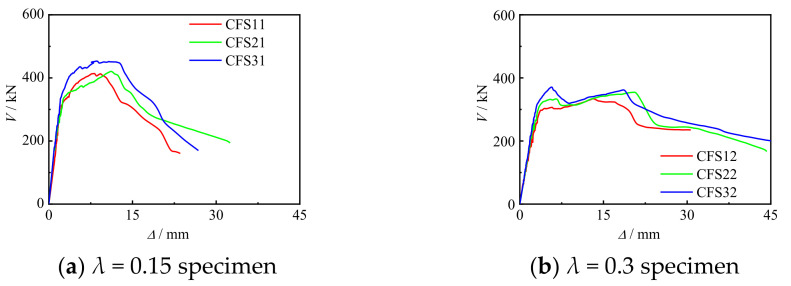

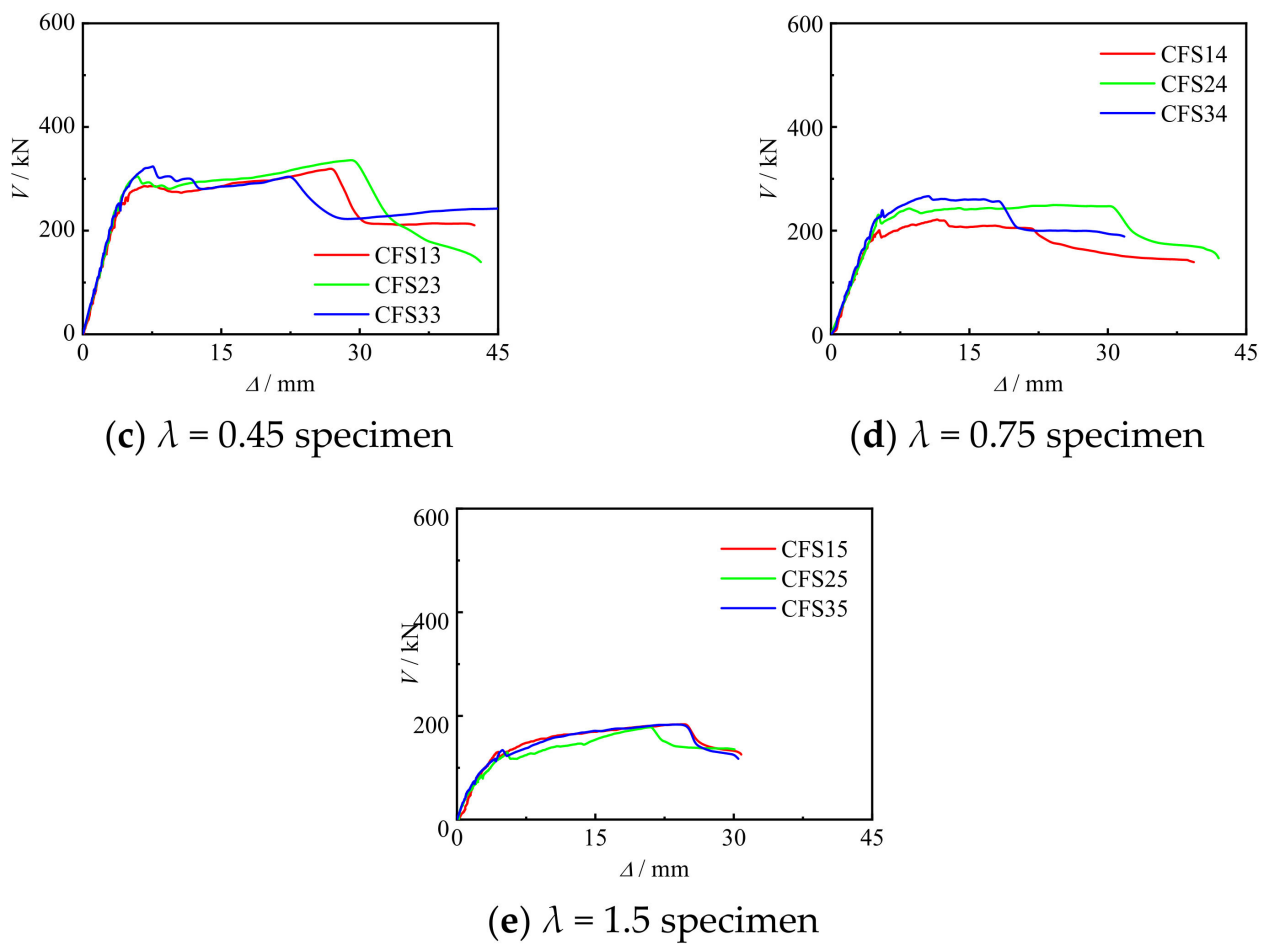
*V*-Δ curve of specimens.

Figure 9 shows the *V*-Δ curve of the specimen. In the early stage of loading, there was a linear relationship between shear loading and displacement, and the stiffness of the curve was relatively high, being in the elastic stage. Afterwards, the test curves of the specimens with different types of failure differed.

For specimens subjected to bending shear failure, when the shear reached approximately 80% of the peak shear loading, the stiffness of the curve decreased. After the longitudinal CFRP was pulled apart, the shear force decreased, and when the shear force reached 80% of the peak shear force, the elastic–plastic stage ended. As the displacement increased, the shear force began to rise; the curve shows a decreasing section after the peak shear force. In addition, as the number of transverse CFRP layers increased, the bearing capacity of the specimen increased, and there was no significant change in the stiffness during the elastic stage of the curve. The main load values of the specimens are shown in Table 5.

#### 3.2.2. Collaborative Performance of Steel Tube and CFRP

**Figure 10:**
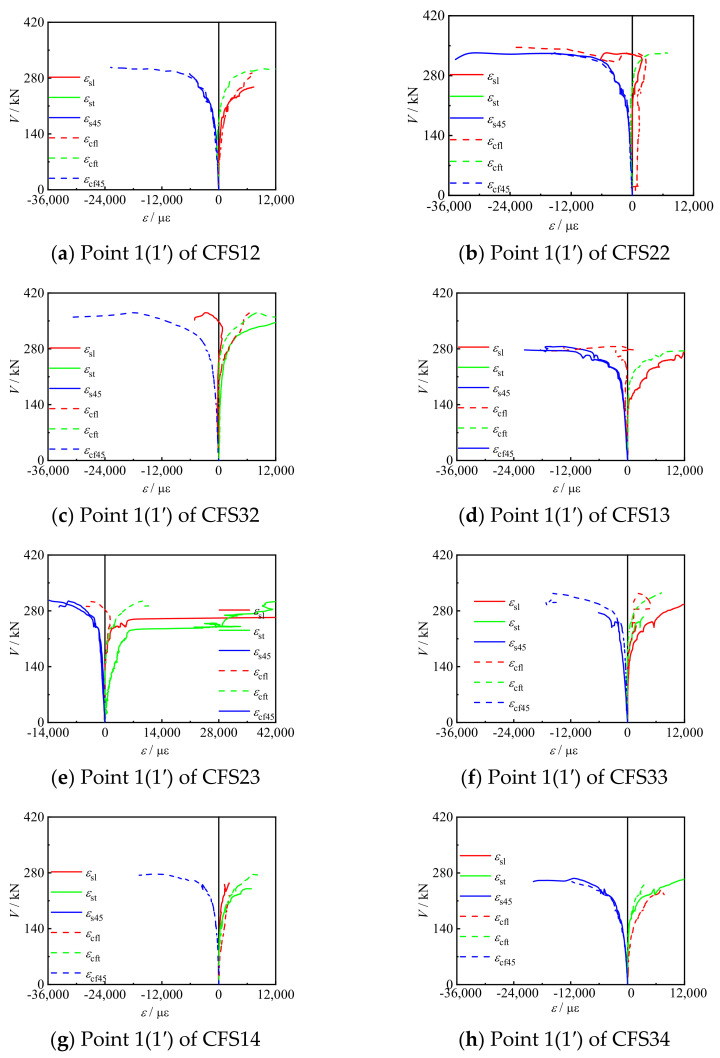
*V*-*ε* curve of specimens.

Figure 10 shows the *V*-*ε* curves of the circular bending shear specimens and square bending shear specimens. *ε*_cf_ and *ε*_s_ were basically consistent, indicating that the two materials can work together.

## 4. Finite Element Simulation

### 4.1. Model of FES

**Figure 11:**
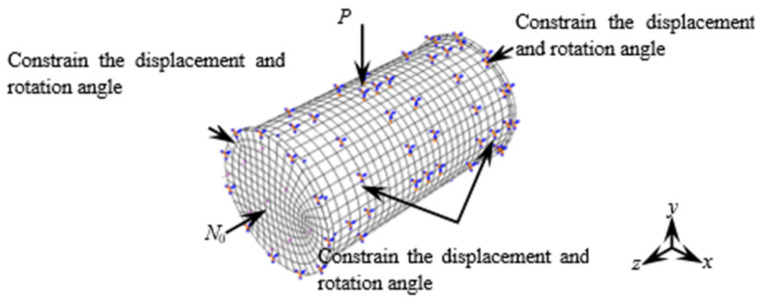
Boundary conditions for FES of bending shear members.

The CFRP concrete-filled steel tube bending–shear members were investigated using a full model calculation; Figure 11 shows the boundary conditions for the finite element simulation of the bending shear specimens [26,27,28]. A solid element simulation (C3D8R) was conducted on the steel tube, concrete, and end plates, which was a linear brick element with eight nodes to reduce integration. The CFRP adopted an M3D4R membrane structure. In addition, Von Mises stress was chosen as the criterion for the yield stage. The CFRP was only subjected to tensile stress; therefore, Hooke’s law was used for calculations before its fracture. When *ε*_cftr_ and *ε*_cflr_ were reached, it indicated that the CFRP had failed due to fractures. Due to the compressive and non-tensile properties of the concrete, the ultimate tensile stress of the concrete (6.752 Mpa) was used as the failure criterion. The tensile strength of the steel pipes (*f*_u_ = 610 Mpa) was used as the failure criterion. As the pressure between the steel tube and concrete could only be transferred on the surfaces of two materials, the hard contact mode was adopted between them. In the finite element simulation, there was no slip in the tangential direction, and there was hard contact between the end plate and the concrete in the normal direction. According to the test results in Figure 10, it can be seen that the strains of the CFRP and steel tube remained consistent. Therefore, it is assumed that there is no slipping between the CFRP and steel tube, and that the two contact adopted bonding. The geometry of the specimens and mesh type is shown in Table 6.

### 4.2. Comparison between Simulation Results and Experimental Results

#### 4.2.1. Shear–Displacement Curve

**Figure 12:**
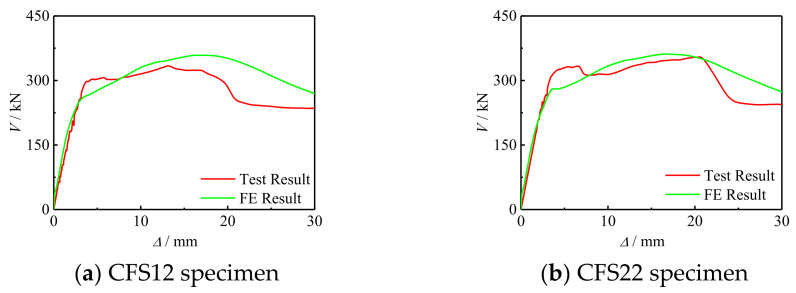

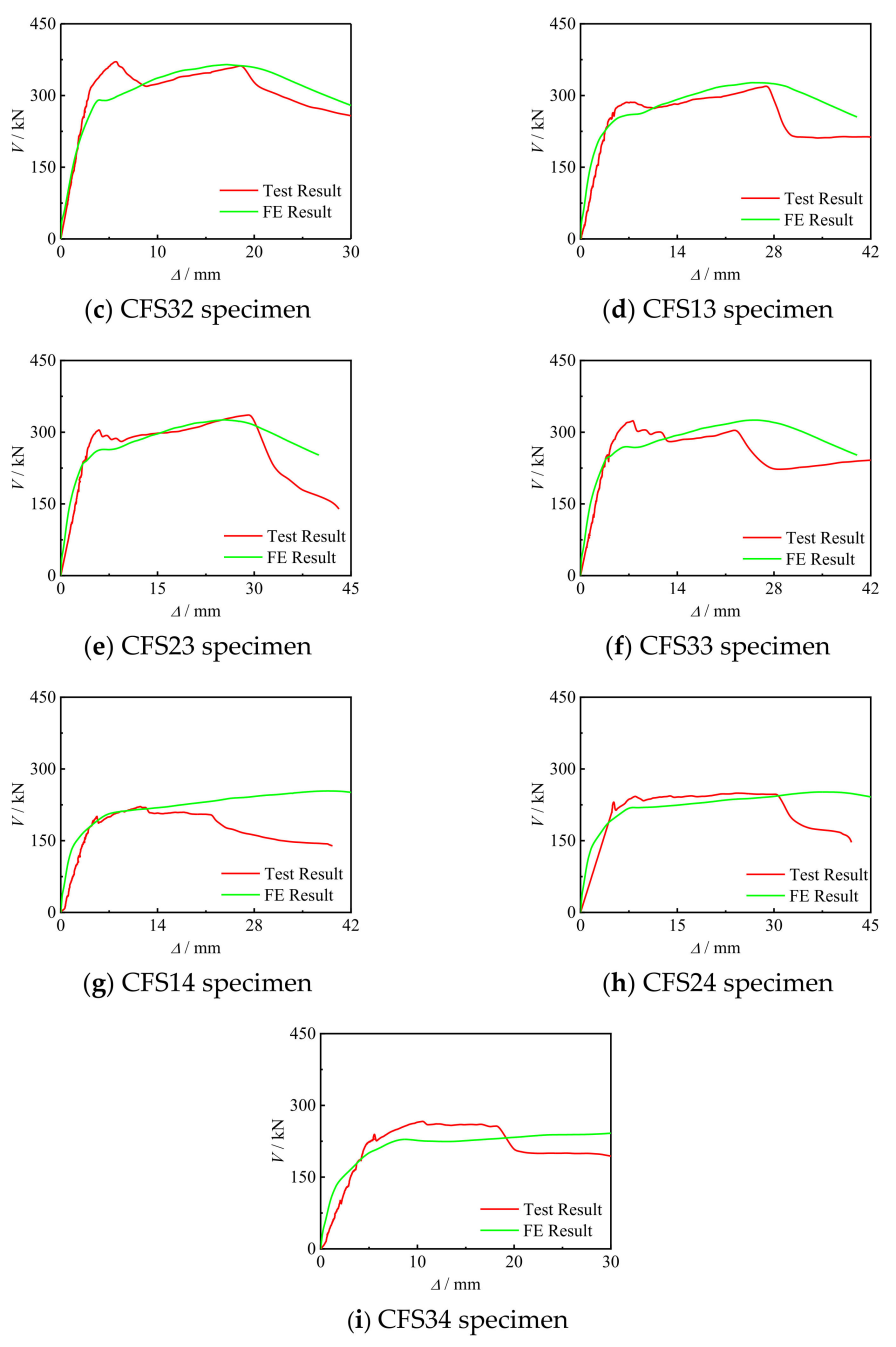
Comparison of *V*-Δ curve simulation results and experimental results of the specimens.

Figure 12 shows the comparison between the simulation results of the *V*-Δ curve of the bending shear specimen and the experimental results, calculating the elastic stage stiffness and bearing capacity of 15 short columns of test and FE curves, with an average error of 6.71% and a mean square error of 0.83 for the elastic stage stiffness. The average error of the bearing capacity was 3.88%, with a mean square error of 0.94.

#### 4.2.2. Failure Modes

**Figure 13:**
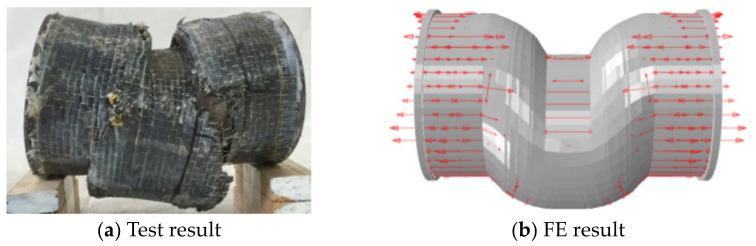
The failure modes of CFS14. Arrows in the figure indicate the whole CFRP that have not yet broken.

**Figure 14:**
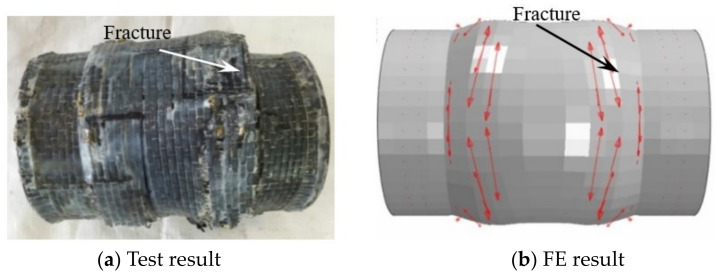
The failure modes of transverse CFRP of CFS22. Arrows in the figure indicate the transverse CFRP that have not yet broken.

**Figure 15:**
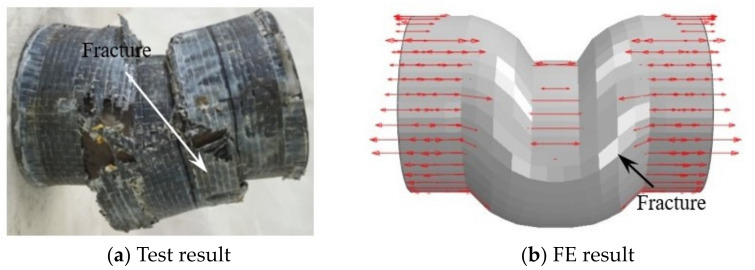
The failure modes of the longitudinal CFRP of CFS34. Arrows in the figure indicate the longitudinal CFRP that have not yet broken.

Figure 13, Figure 14 and Figure 15, respectively, show the failure mode of the CFS14, CFS22, and CFS34 circular bending shear specimens (arrows in the figure indicate the transverse and longitudinal CFRP that have not yet broken). There was a certain bending deformation in the loading area in test results, which was different from the FE results. This was due to the influence of the loading equipment during the test process, but the crack location of the CFRP in the test result and FE result was basically the same.

### 4.3. Full-Process Analysis of Stress

#### 4.3.1. Typical V-D Curve

**Figure 16:**
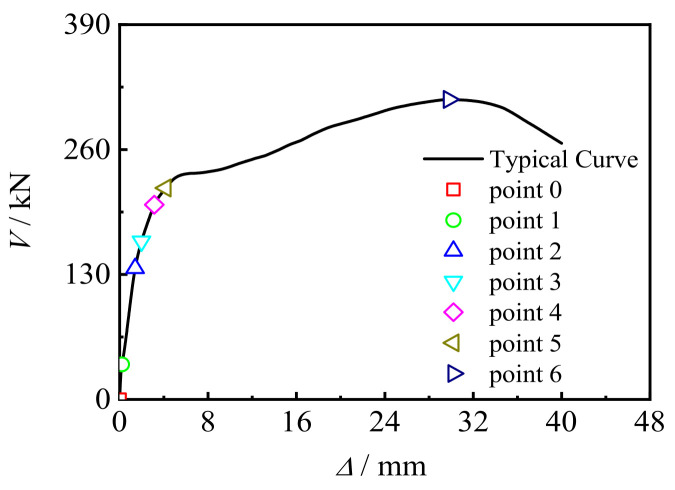
The typical *V*-Δ curve.

Figure 16 shows the typical *V*-Δ curve of the CFRP concrete-filled steel tube bending shear members. Six characteristic points were selected: point 1 (0 mm,0 kN) corresponds to the proportional limit of stress, point 2 (1.03906 mm, 107.9402 kN) corresponds to the yield of the steel, point 3 (1.99573 mm, 165.22152 kN) corresponds to the fracture of the longitudinal CFRP lower tension area, point 4 (2.1759 mm, 171.873 kN) corresponds to concrete cracking, point 5 (3.115 mm, 202.05394 kN) corresponds to the fracture of the transverse CFRP compression area, and point 6 (31.10646 mm, 311.53309 kN) corresponds to the ultimate bearing capacity of the component.

#### 4.3.2. Stress in Concrete

**Figure 17:**
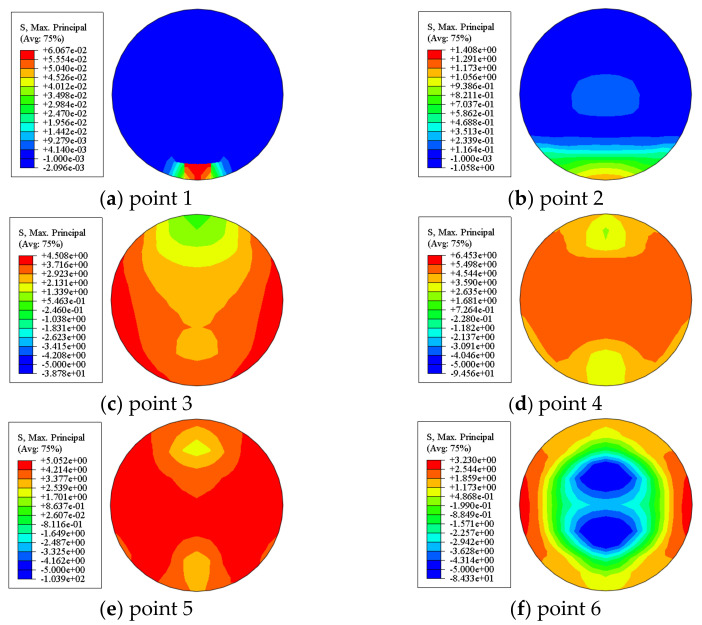
Stress distribution of concrete cross-section in the shear span area.

Figure 17 shows the maximum principal stress distribution of the concrete cross-section in the shear span area of a circular bending shear member. The maximum principal stress of the concrete in the shear span area was symmetrical along the axis of symmetry. At point 1, the entire cross-section of the concrete in the shear span area was under tension. At point 2 of the circular component, as the shear loading gradually increased, the main stress on both sides of the concrete was the highest, and the concrete bore some of the shear loading. At point 3 of the circular component, the maximum tensile stress was reached on the outer edges of both sides of the concrete and the concrete cracked. Afterwards, the maximum principal stress of the concrete gradually decreased, and there were many cracks in the concrete, gradually withdrawing from work. At point 6, compressive stress appeared in the core area of the concrete, and compressive stress also appeared at the corners of the square components. In the initial stage of loading, the concrete in the shear span area was mainly subjected to shear action. In the later stage of loading, the bending deformation of the component was relatively large, and the concrete was mainly subjected to bending action.

#### 4.3.3. Stress in the Steel Tube

**Figure 18:**
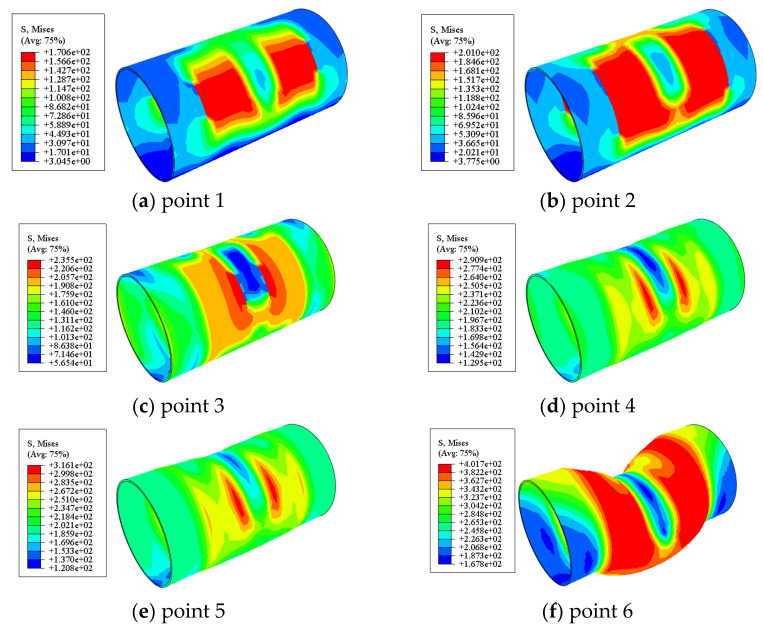
Mises stress distribution of the steel tube in the circular bending shear members.

Figure 18 shows the Mises stress distribution of the steel tube in the circular bending shear members. Due to the small loading, the steel tube was still in the elastic stage. At point 2, both sides of the upper fixture of the circular component first entered the yield stage; afterwards, as the load continued to increase, the yield zone developed towards both ends of the component. At point 6, the load reached its bearing capacity, and the Mises stress of the steel tubes at the upper fixture and lower support was relatively small compared to the other parts. The reason for this was that the steel tubes at the lower support had been sheared out, and the steel tubes at the upper fixture had undergone local buckling and had been damaged. Throughout the entire stress process, the stress of the steel was symmetrically distributed along the middle section.

#### 4.3.4. Stress in the CFRP

**Figure 19:**
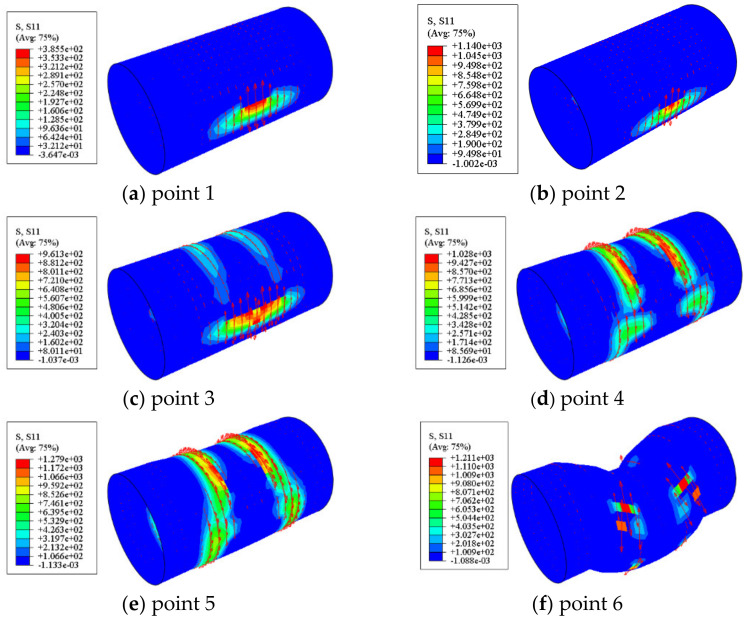
The stress distribution of the transverse CFRP in the circular bending shear members. Arrows in the figure indicate the transverse CFRP that have not yet broken.

Figure 19 shows the stress distribution of the transverse CFRP in the circular bending shear members. The lateral CFRP played a restraining role due to the local buckling deformation of the steel tubes on both sides of the fixture. Component fracturing occurred on both sides of the fixture, and the tensile stress value reached an extreme value at point 5, ultimately breaking at both sides of the fixture in the shear span zone. At point 6, almost all of the transverse CFRP within the shear span area of the circular component fractured, providing greater restraint for the component in the shear span area.

## 5. Calculating Expressions and Expression Validation

**Figure 20:**
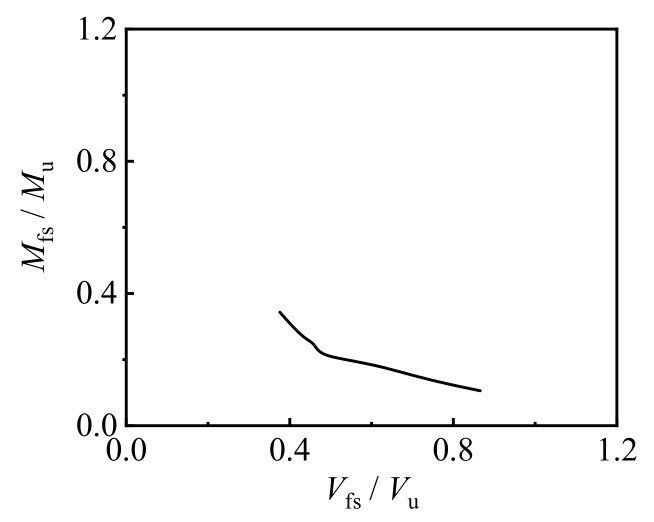
Typical *M*_fs_*/M*_u_—*V*_fs_*/V*_u_ correlation curve for the bending shear members.

The use of the finite element calculation method can accurately calculate the load displacement curve of concrete-filled CFRP steel tubes under a bending–shear load, which is conducive to in-depth research on its mechanical properties. However, the calculation is complex and not conducive to practical engineering applications. Therefore, it is necessary to provide a simplified calculation method based on experiments and finite element analysis. Figure 20 shows a typical *M*_fs_*/M*_u_—*V*_fs_*/V*_u_ correlation curve for CFRP concrete-filled steel tube bending–shear members, where *M*_fs_ represents the flexural bearing capacity of the bending shear member and *V*_fs_ represents the shear-bearing capacity of the bending shear member [29,30].

By conducting substantial parameter calculations, the calculation parameters (applicable range) were: *f*_y_ = 235–390 Mpa, *f*_cu_ = 30–90 Mpa, *a* = 0.05–0.2, *ξ*_cf_ = 0~0.8, *η*_cf_ = 0–0.9, *λ* = 0.3~0.75, *ξ* = 0.4–1.6. By fitting the experimental results with the finite element results, we obtained the following relevant equations:

(*V*_fs_/*V*_u_)^0.2^ + *a*(*M*_fs_/*M*_u_)^1.37^ = 1 (1)

*a* = (*η*^0.11^ − 2.02)/*ξ*^15.9^ (2)

*γ* = 15,000 + 8808(0.2 − *λ*) (3)

When *γ* reaches Equation (3), the specimen reaches its shear-bearing capacity.

**Figure 21:**
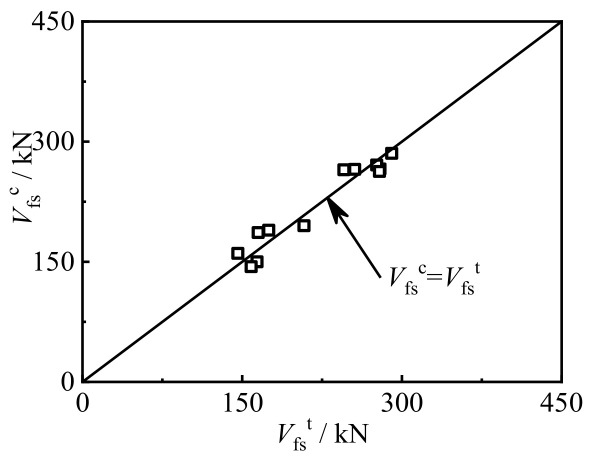
Comparison of *V*_fs_^c^/*V*_fs_^t^ for CFRP concrete-filled steel tube bending–shear members.

In order to verify the rationality of the obtained bearing capacity calculation formula, the calculation results were compared with the experimental results. Figure 21 shows the comparison between the calculated bearing capacity *V*_fs_^c^ and the experimental value *V*_fs_^t^ of the CFRP concrete-filled steel tube bending–shear specimen. The average value of *V*_fs_^c^/*V*_fs_^t^ for the circular components was 0.91, with a mean square error of 0.21.

## 6. Conclusions

Static tests were conducted on 15 circular concrete-filled CFRP steel tube bending–shear specimens. The shear displacement (*V*-Δ) curve of the specimen and the collaborative work between the steel tube and CFRP were discussed. ABAQUS was applied to simulate the *V*-Δ curve and the failure mode of the specimens. We explored the effects of CFRP layers, material strength, the steel ratio, and the shear span ratio on the bending–shear performance of the components. The moment shear correlation equation for concrete-filled CFRP steel tube bending–shear members was presented. The main conclusions are presented as follows:

1. According to the shear span ratio, the shear failure of the specimens can be divided into pure shear failure (*λ* = 0.15), bending shear failure (*λ* = 0.3~0.75), and bending failure (*λ* = 1.5).
2. Steel tubes and CFRP can work together; as the shear span ratio increases, the bearing capacity and stiffness of the specimen decrease; while an increase in the number of transverse CFRP layers can improve the bearing capacity, it has no significant effect on stiffness.
3. ABAQUS was applied to simulate the *V*-Δ curve and failure mode of the bending–shear specimens, and the simulation results were in good agreement with the experimental results.
4. The relevant equation for the bearing capacity of the bending shear members was given, and the calculation results using this equation were in good agreement with the experimental results.
5. The coupling effects of the compression bending–shear loads of concrete-filled CFRP steel tubes should be conducted in conditions that are closer to actual working conditions in the future.

## Figures and Tables

**Table 1:** Main parameters of specimens.

No.	*L*/mm	*λ*	*m*_t_/Layer	*ξ*_ss_	*ξ*_cf_	*ξ*
CFS11	66	0.15	1	1.06	0.16	1.22
CFS12	102	0.3	1	1.06	0.16	1.22
CFS13	138	0.45	1	1.06	0.16	1.22
CFS14	210	0.75	1	1.06	0.16	1.22
CFS15	390	1.5	1	1.06	0.16	1.22
CFS21	66	0.15	2	1.06	0.32	1.38
CFS22	102	0.3	2	1.06	0.32	1.38
CFS23	138	0.45	2	1.06	0.32	1.38
CFS24	210	0.75	2	1.06	0.32	1.38
CFS25	390	1.5	2	1.06	0.32	1.38
CFS31	66	0.15	3	1.06	0.49	1.54
CFS32	102	0.3	3	1.06	0.49	1.54
CFS33	138	0.45	3	1.06	0.49	1.54
CFS34	210	0.75	3	1.06	0.49	1.54
CFS35	390	1.5	3	1.06	0.49	1.54

**Table 2:** Performance of steel tubes used in C-CF-CFRP-ST specimens.

*f*_y_/MPa	*f*_u_/MPa	*E*_s_/GPa	*v*_s_	*e*’/%
466	610	206	0.28	27

**Table 3:** Mix proportion of concrete used for C-CF-CFRP-ST specimens.

Cement	Fly Ash	Sand	Gravel	Water
0.6	0.4	2	1.4	0.35

**Table 4:** Main mechanical property indexes of CFRP.

Thickness (mm)	*E*_cf_ (GPa)	*ε*_cftr_ (μe)	*ε*_cflr_ (μe)
0.111	230	5500	5500

**Table 5:** Main load values of specimens.

No.	Displacement at Yielding/mm	Yielding Load/kN	Displacement at Maximum Load/mm	Maximum Load/kN
CFS11	4.93	337.61	8.22	415
CFS12	5.5	305.45	12.97	333.65
CFS13	6.13	283.9	27.78	311.2
CFS14	6.93	184.63	11.2	211.9
CFS15	5.98	132.88	25.98	183.54
CFS21	6.43	374.5	11.03	420.55
CFS22	7.39	302.7	20.46	354.25
CFS23	7.59	291.8	30.08	319.6
CFS24	7.06	211.9	32.2	258.16
CFS25	6.09	134.69	18.19	185.41
CFS31	6.08	429.9	452.3	8.32
CFS32	7.34	318.8	5.29	365.15
CFS33	7.98	307.1	7.46	322.46
CFS34	6.98	223.6	19.32	277.36
CFS35	6.32	128.71	26.13	188.36

**Table 6:** Geometry of the specimens and mesh type.

Geometry	Steel	Concrete	CFRP	End Plate
Section (mm)	120 × 120	116 × 116	Different dimensions	200 × 200
Thickness (mm)	2	/	Different dimensions	20
Length (mm)	Different	Different	Different	/
Type of geometry	Solid	Solid	Shell	Solid
Mesh type	C3D8R	C3D8R	M3D4	C3D8R

## Data Availability

The raw data supporting the conclusions of this article will be made available by the authors on request.
